# Detection of hand motion during cadaveric mastoidectomy dissections: a technical note

**DOI:** 10.3389/fsurg.2024.1441346

**Published:** 2024-10-03

**Authors:** Thomas J. On, Yuan Xu, Nicolas I. Gonzalez-Romo, Gerardo Gomez-Castro, Oscar Alcantar-Garibay, Marco Santello, Michael T. Lawton, Mark C. Preul

**Affiliations:** ^1^ The Loyal and Edith Davis Neurosurgical Research Laboratory, Department of Neurosurgery, Barrow Neurological Institute, St. Joseph’s Hospital and Medical Center, Phoenix, AZ, United States; ^2^ School of Biological and Health Systems Engineering, Arizona State University, Tempe, AZ, United States

**Keywords:** artificial intelligence, surgical motion analysis, machine learning, deep learning, neural networks, neurosurgery

## Abstract

**Background:**

Surgical approaches that access the posterior temporal bone require careful drilling motions to achieve adequate exposure while avoiding injury to critical structures.

**Objective:**

We assessed a deep learning hand motion detector to potentially refine hand motion and precision during power drill use in a cadaveric mastoidectomy procedure.

**Methods:**

A deep-learning hand motion detector tracked the movement of a surgeon's hands during three cadaveric mastoidectomy procedures. The model provided horizontal and vertical coordinates of 21 landmarks on both hands, which were used to create vertical and horizontal plane tracking plots. Preliminary surgical performance metrics were calculated from the motion detections.

**Results:**

1,948,837 landmark detections were collected, with an overall 85.9% performance. There was similar detection of the dominant hand (48.2%) compared to the non-dominant hand (51.7%). A loss of tracking occurred due to the increased brightness caused by the microscope light at the center of the field and by movements of the hand outside the field of view of the camera. The mean (SD) time spent (seconds) during instrument changes was 21.5 (12.4) and 4.4 (5.7) during adjustments of the microscope.

**Conclusion:**

A deep-learning hand motion detector can measure surgical motion without physical sensors attached to the hands during mastoidectomy simulations on cadavers. While preliminary metrics were developed to assess hand motion during mastoidectomy, further studies are needed to expand and validate these metrics for potential use in guiding and evaluating surgical training.

## 1 Introduction

Manual dexterity, strong neuroanatomical knowledge, and proficient use of instruments and the surgical microscope are fundamental to successful neurosurgical procedures in the operating room. Mastoidectomy stands out as a demanding microsurgical procedure requiring synchronized and controlled high-speed drilling to achieve sequential exposure of delicate anatomical structures during temporal bone dissection (1, 2).

During bone removal, critical neurovascular structures, such as the facial nerve, sigmoid sinus, and inner ear structures, must be protected. This procedure is done with millimetric precision, especially as the depth increases (3). For trainees, practicing surgical techniques with cadaveric simulation helps them acquire, develop, and refine their surgical skills and confidence with the drill, microscope, and microsurgical instruments (3–5).

Computer-based assessments, including haptic-feedback devices and virtual reality simulators, have been developed to quantitatively evaluate surgical performance in mastoidectomy training (6–9). The surgical instruments of users during mastoidectomy have also been tracked using video analysis and computer vision (10, 11). However, technology based on deep learning has yet to be used to track hand motion during mastoidectomy simulation.

In this technical note, we used a convolutional neural network trained to detect hand motion during three cadaveric mastoidectomy procedures. We aim to evaluate this technology as a potential future surgical performance assessment tool in a cadaver laboratory setting during mastoidectomies.

## 2 Methods

**Figure 1:**
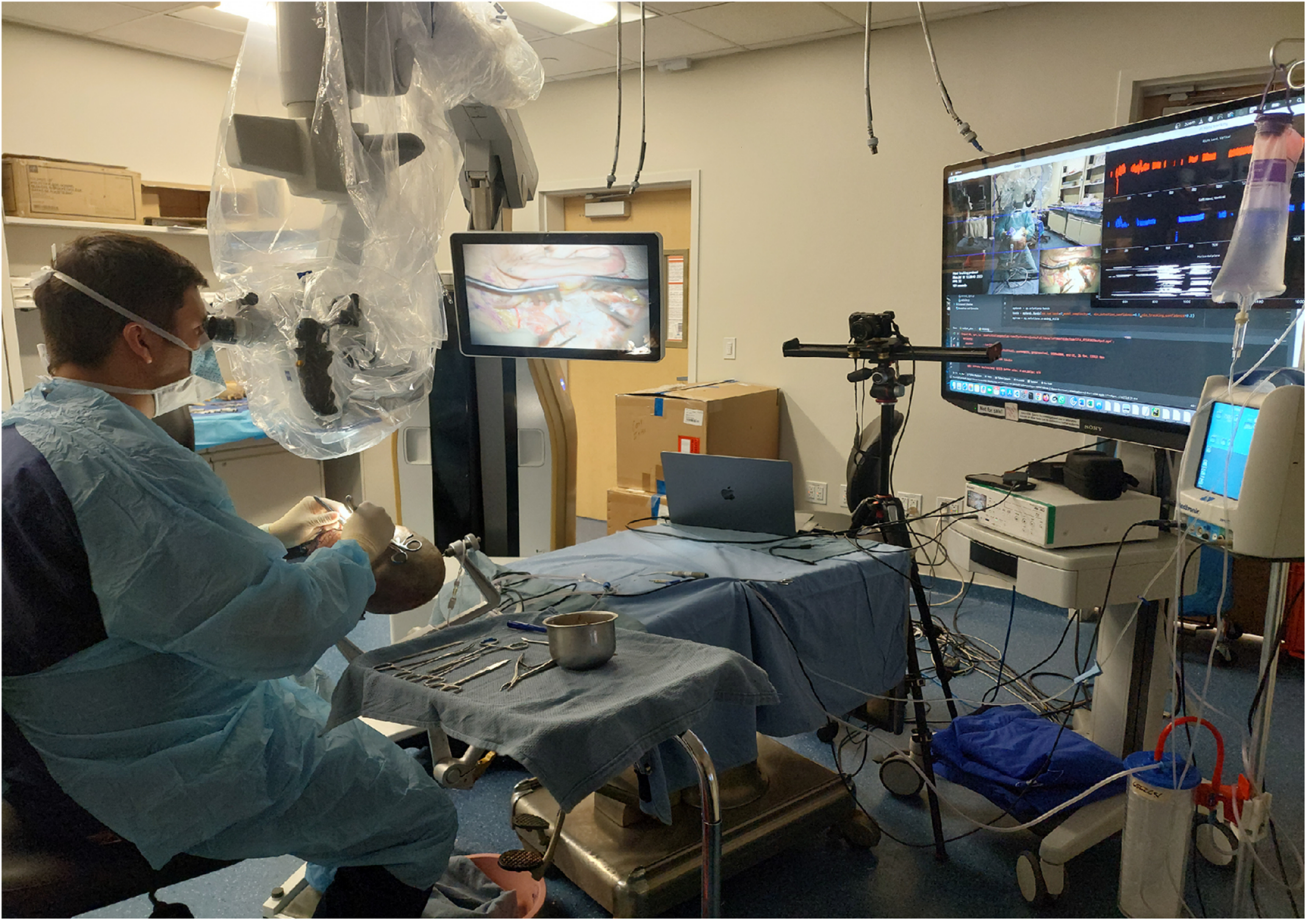
The setup for hand tracking during mastoidectomy is shown. The instrument table is positioned to the user's right, the microscope to the left, and the camera 1.5 m in front of the surgeon. Real-time tracking is displayed on the screen to the user's right. Used with permission from Barrow Neurological Institute, Phoenix, Arizona.

Mastoidectomies were performed using three cadaveric head specimens fixed using a Mayfield holder device with the head in the lateral position. A Zeiss Kinevo surgical microscope (Carl Zeiss AG, Jena, Germany) was used for visualization, and standard microsurgical instrumentation, including a high-speed drill, was used for the dissection. Gloves and a surgical gown were worn to simulate operating room conditions (Figure 1).

### 2.1 Surgical technique

Burrs of different sizes were used during various stages of the mastoidectomy procedure [4 or 3 millimeter (mm) cutting burrs and 2 mm diamond burrs] (12) under continuous saline irrigation. External landmarks were identified, including the spine of Henle, mastoid tip, temporal line, and Macewen's triangle. Using a 4 mm cutting burr, a kidney-shaped cavity was drilled, removing the cancellous bone of the mastoid air cells. The sigmoid sinus, sinodural angle, and middle fossa plate were exposed. The mastoid antrum was entered, and the incus was identified. Under continuous irrigation, the remaining air cells were removed, exposing the fallopian canal, semicircular canals, presigmoid dura, and endolymphatic duct. At the infralabyrinthine space, the jugular bulb was exposed.

### 2.2 Hand motion detection

Mastoidectomy simulations were captured by a camera (Sony A6000 camera, Sony Corp., Tokyo, Japan) mounted on a tripod positioned 1.5 m in front of the surgeon. The video output was processed by a deep learning hand motion detector to determine 21 hand landmarks corresponding to digit joints and wrists of both hands. This technology is built upon an open-source convolutional neural network (13, 14) (MediaPipe, https://ai.google.dev/edge/mediapipe/solutions/vision/hand_landmarker).

The video input was arranged in a picture-in-picture format to simultaneously capture vertical and horizontal surgical hand motion and the microsurgical operative view. A blue-line 3 × 3 grid with nine cells was designed for calibration: the hands were positioned in the center cell (cell 5), the instrument table in the middle left cell (cell 4), and the microscope handles in the top three cells (cells 1, 2, and 3). The microsurgical video feed was placed in the bottom right cell (cell 9). A timestamp was included in the video recording to facilitate correlation analysis with the tracking data. Detection of landmark 12, corresponding to the tip of the third digit of each hand, was used to calculate the time spent in each cell for both hands.

The deep learning motion detection model produced a time series of landmarks corresponding to both hands' horizontal and vertical coordinates. This data was later used to create tracking plots using the matplotlib library (https://matplotlib.org/) and perform statistical analysis using the pandas library (https://pandas.pydata.org/), both of which are Python libraries (Python 3.11, Python Software Foundation, https://www.python.org/). Continuous variables were reported as mean (SD).

## 3 Results

### 3.1 Descriptive analysis

1,948,837 landmarks were detected during 30 min of recordings (10 min per procedure), translating to an overall detection performance of 85.9%. 939,540 (48.2%) landmark detections corresponded to the right hand (dominant). 1,007,916 (51.7%) detections corresponded to the left hand (non-dominant), and 1,381 (0.1%) were null detections (Table 1).

**Table 1:** Hand motion detection performance during cadaveric mastoidectomy procedures.

Procedure	Number of detections	Detection performance	Right hand detections	Left hand detections	Null detections
1	621,898	82.2%	345,786 (55.6%)	275,646 (44.3%)	466 (0.7%)
2	641,625	84.8%	254,583 (39.6%)	386,757 (60%)	285 (0.4%)
3	685,314	90.6%	339,171 (49.4%)	345,513 (50.4%)	630 (0.1%)
Total	1,948,837	85.9%	939,540 (48.2%)	1,007,916 (51.7%)	1,381 (0.1%)

**Figure 2:**
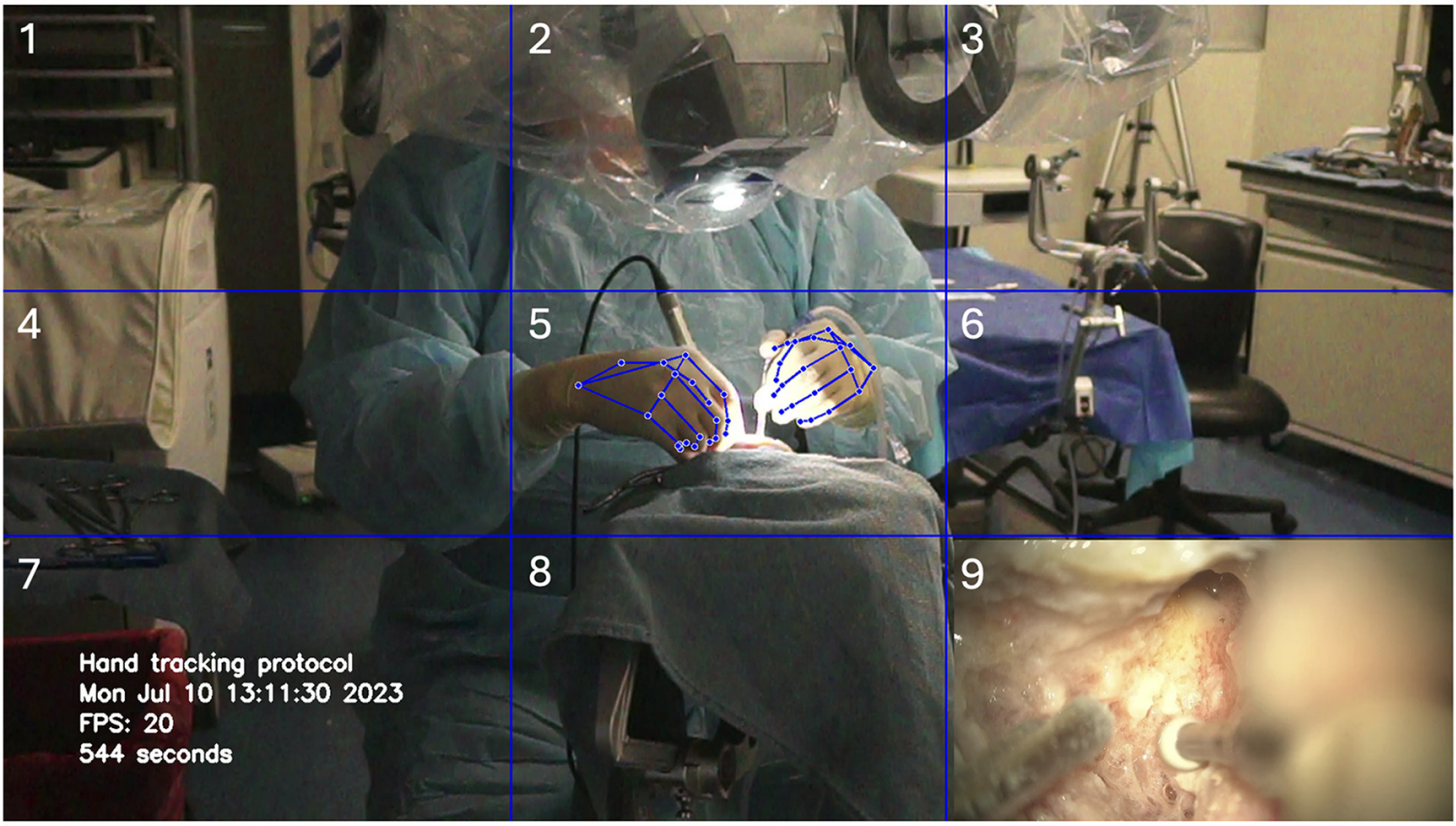
Deep learning hand motion detection during cadaveric mastoidectomy. A 3 × 3 grid was created with nine cells delimited by horizontal and vertical blue gridlines. Used with permission from Barrow Neurological Institute, Phoenix, Arizona.

**Figure 3:**
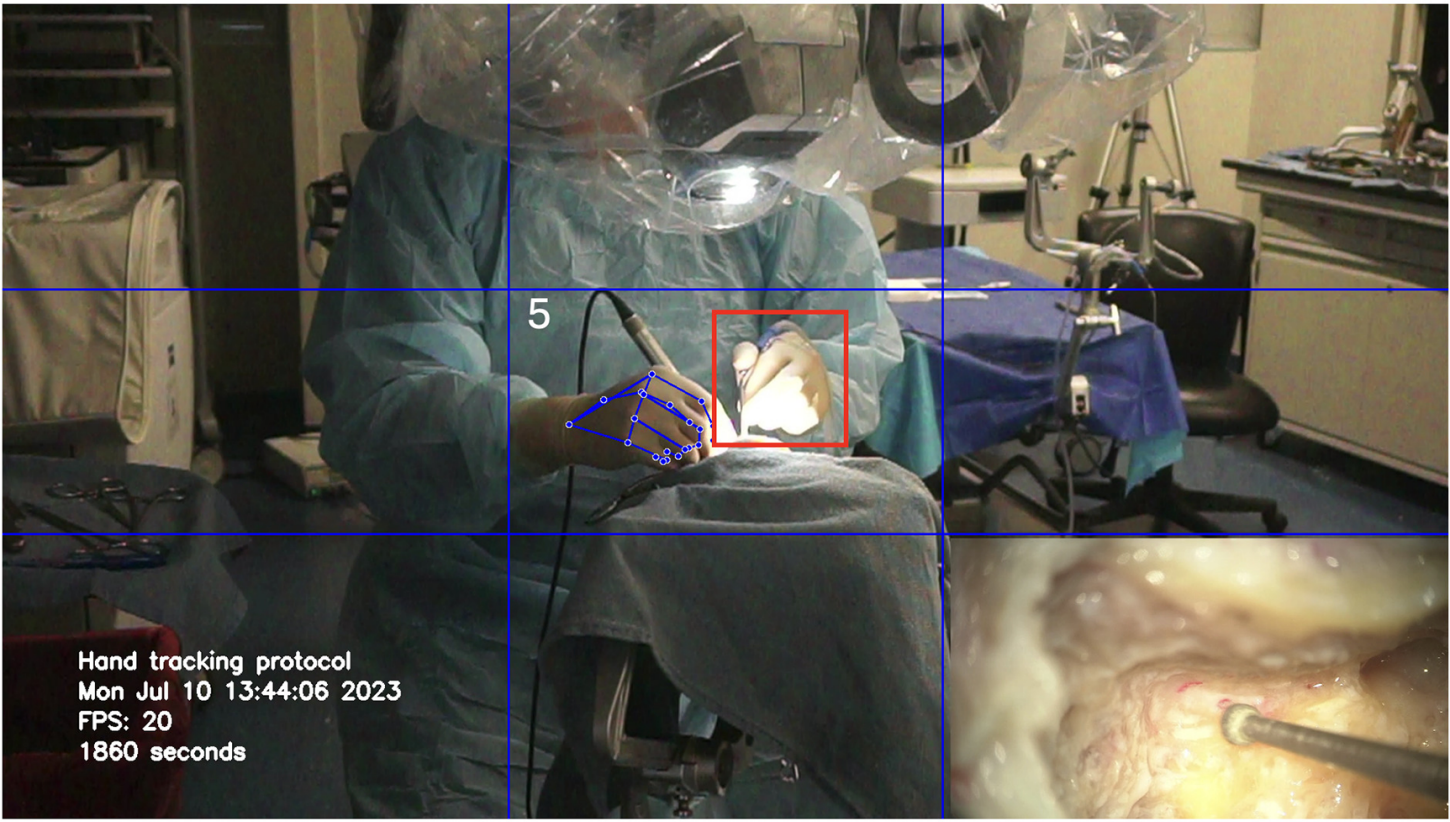
Loss of tracking of the non-dominant hand (red square) due to the increased brightness at the center of the image. Used with permission from Barrow Neurological Institute, Phoenix, Arizona.

Hand motion detection was possible throughout every part of the surgical procedure, including skin incision, drilling (Figure 2), dissection of anatomical structures, adjusting zoom/focus using the microscope handle controls, and changing instruments. The bone dust produced during the drilling did not alter hand detection. Loss of tracking occurred because of the increased microscope light at the center of the surgical field (Figure 3).

### 3.2 Validation of tracking detection

**Figure 4:**
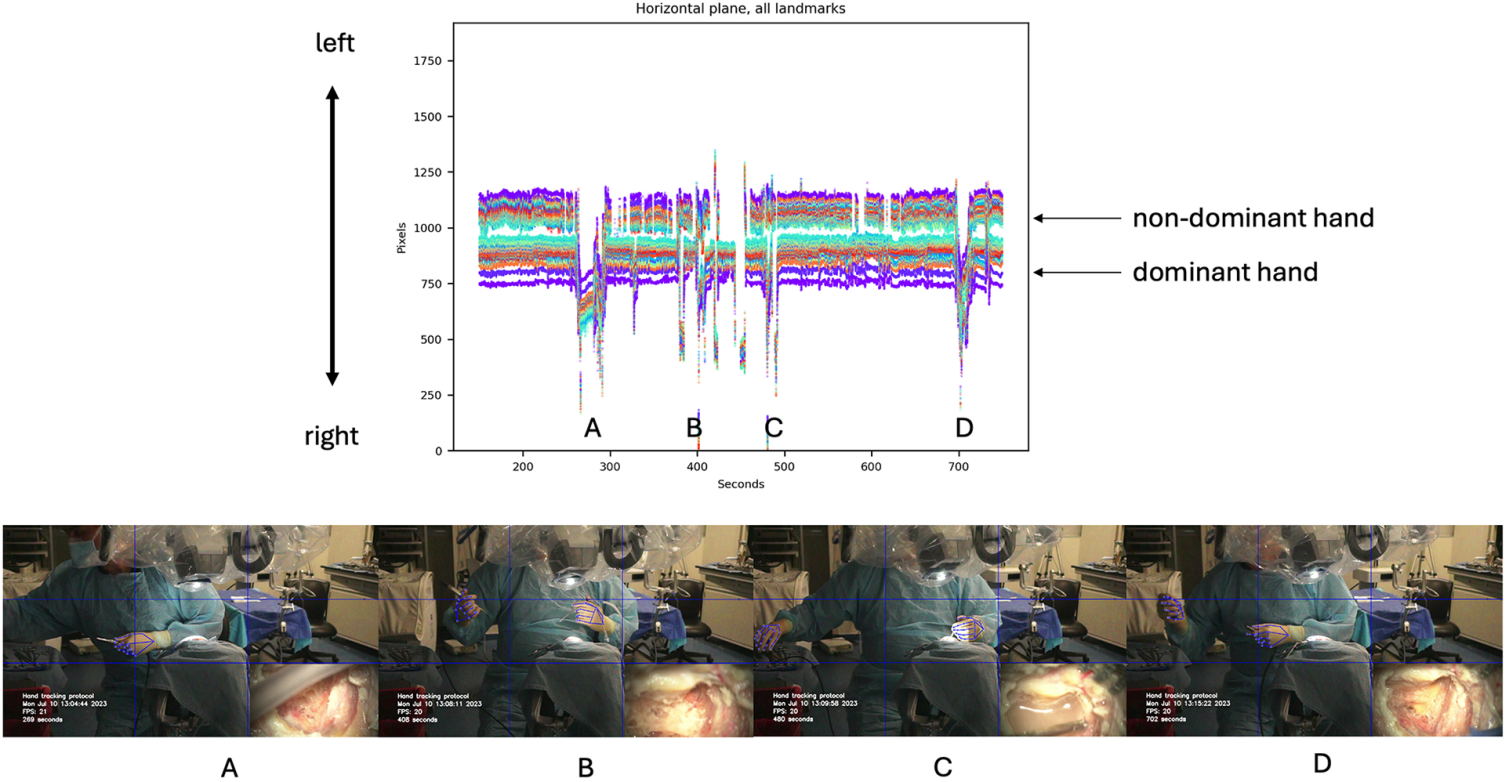
Horizontal motion detection during cadaveric mastoidectomy. Top row: Horizontal tracking plot for both hands. Bottom row: Large amplitude spikes correspond to changes in instruments, as shown in the video frames for each detection spike **(A–D)**. Used with permission from Barrow Neurological Institute, Phoenix, Arizona.

Horizontal motion data of both hands during the first mastoidectomy were analyzed and graphed, revealing significant spikes in the dominant hand channel indicating instrument changes, specifically when changing drill bits between drilling (Figure 4). Cutting burrs were used for drilling cancellous bone of mastoid air cells, while diamond burrs were employed in later stages to drill compact bone overlying critical structures such as the facial nerve, sigmoid sinus, or dura. Several burr head changes were necessary during the procedure.

**Figure 5:**
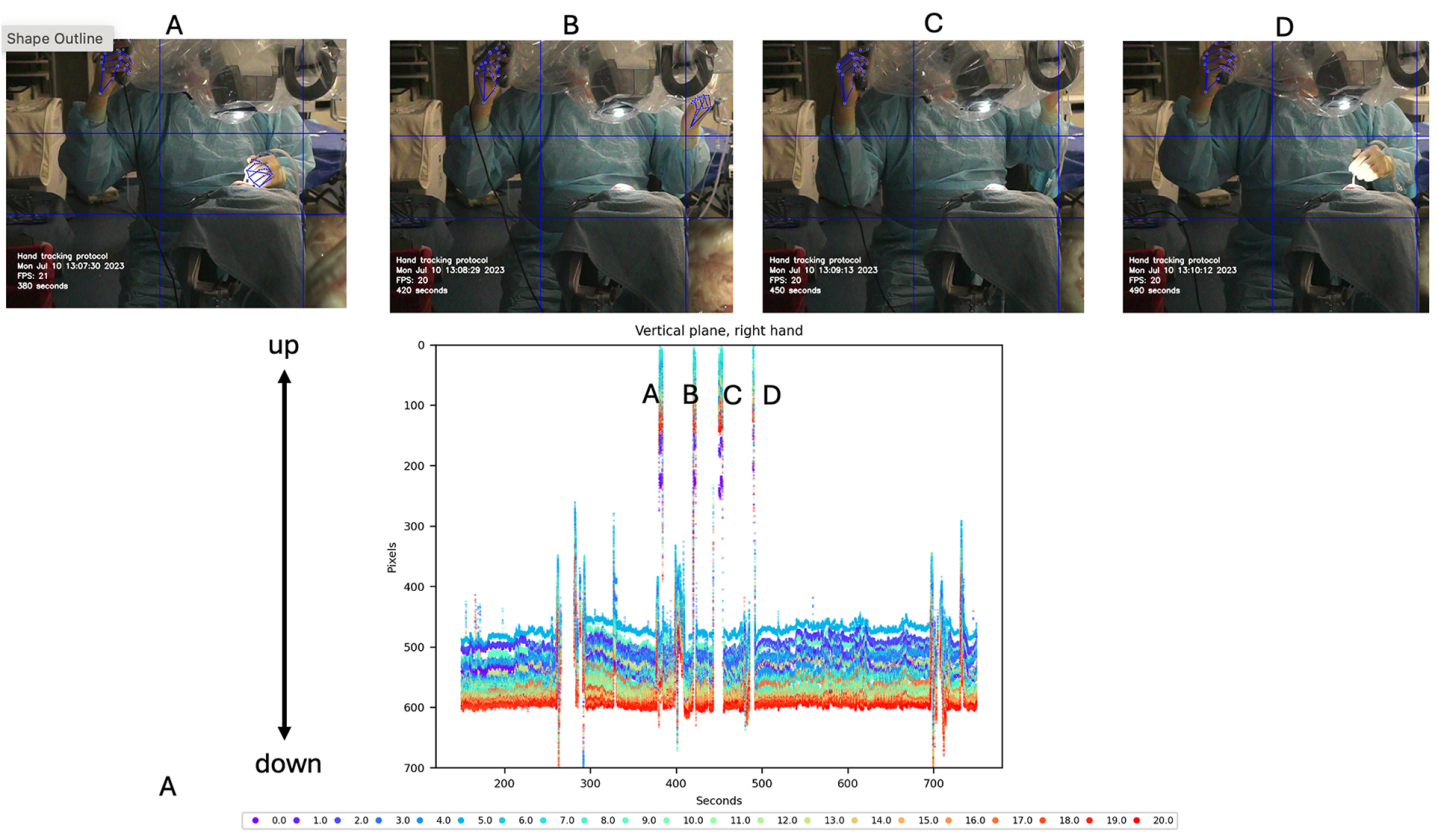
Vertical motion detection of the dominant hand during cadaveric mastoidectomy. Top row: Video frames corresponding to vertical detections observed in the tracking plot. These detections are produced by adjusting the microscope using the handle controls. Bottom row: Vertical tracking plot of hand motion during procedure. Large amplitude spikes are shown that reach out to 0 pixels (top of the image). Each detection spike **(A–D)** is correlated with the specific video frame on the top. Used with permission from Barrow Neurological Institute, Phoenix, Arizona.

Vertical motion data of the dominant hand were also analyzed and graphed, showing large spikes corresponding to movements to reach the microscope handle. This vertical motion was associated with necessary adjustments to the microscope. As the mastoidectomy progressed and deeper structures were reached, adjustments to the microscope's zoom, focus, and positioning were required to maintain optimal visualization. The plot indicated a total of four such adjustments (Figure 5).

### 3.3 Analysis of tracking data

**Figure 6:**
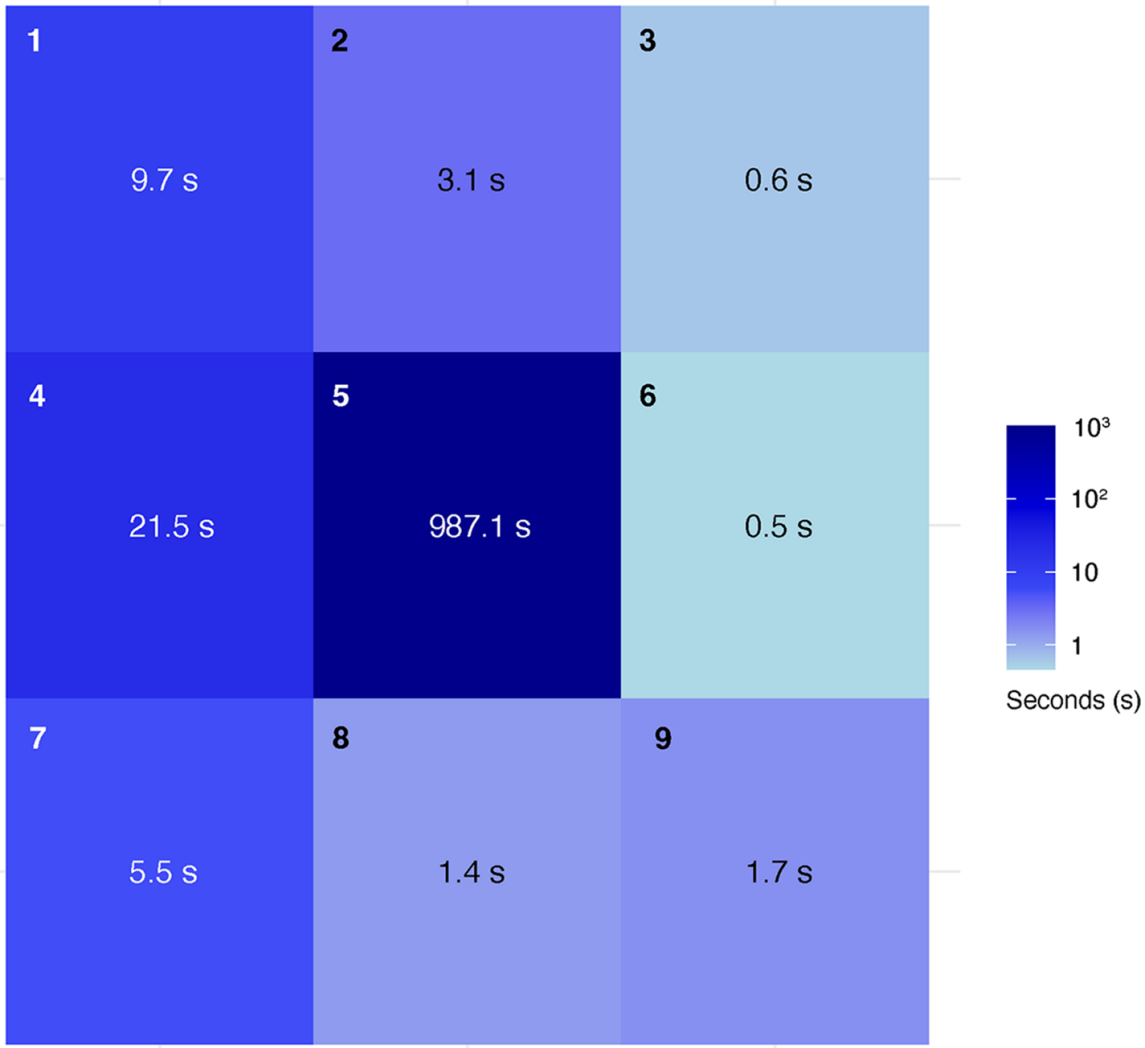
Heatmap showing where hands were positioned in the 3 × 3 grid during the mastoidectomy and the average amount of time (seconds) spent in each cell over three procedures. Used with permission from Barrow Neurological Institute, Phoenix, Arizona.

The hands were tracked to calculate the time spent in each cell for both hands during the procedures. The procedure was performed three times, and the mean (SD) time (seconds) spent within each cell across these procedures was recorded. The hands spent most of the time in cell 5 (centered in the surgical field), with a mean duration of 987.1 (62.7). Movement of the hand to change instruments was primarily detected in cell 4, where the instrument table was located, with a mean duration of 21.5 (15.3). Adjustments to the microscope's position or zoom/focus were detected in cells 1, 2, and 3, near the microscope location, with mean durations of 9.7 (8.6) and 3.1 (2.6), respectively. Additional activity was noted in cell 7, where the trash bin was located, with a mean duration of 5.5 (9.1). Cells 3, 6, 8, and 9 showed minimal movement, with mean durations of 0.6 (1.0), 0.5 (0.5), 1.4 (2.1), and 1.7 (2.9), respectively. The relative time both hands spent in each cell was shown in a heat map (Figure 6).

## 4 Discussion

Mastoidectomy requires recognition of specific anatomical landmarks exposed sequentially during the continuous removal of cancellous and compact bone using a high-speed drill. Protecting the facial nerve and the sigmoid sinus during bone removal is critical, making this procedure a challenging educational task for improving microsurgical drilling skills.

Unlike virtual reality simulators and 3D-printed models, nonpreserved cadaveric bone has anatomy and texture that closely mimic those of actual patients. For learning the mastoidectomy approach, practice on cadaveric tissue offers trainees a realistic simulation to build confidence and precision with the drill, allowing them to skeletonize structures accurately. However, a productive laboratory session requires expert supervision to identify and correct errors (9). In situations where expert feedback is not available, introducing a quantitative method to assess technical skills offers a useful alternative for interpreting performance during mastoidectomy.

Deep learning convolutional neural networks, specialized in visual detection, are appealing for assessing surgical performance as they do not require physical sensors on the surgeon's hands. By detecting hand landmarks corresponding to digit joints and the wrists of both hands, this method also has the advantage of generating large amounts of data for calculating performance metrics. This technology has been used in quantitative assessments during microanastomosis simulations and neuronavigation configuration for burr-hole placement and anatomical landmark selection, recording pre-operative and intra-operative data (15, 16).

As a proof-of-concept study, we used a deep learning model to detect hand motions during mastoidectomy procedures. The deep learning model measured an average of 1,082 landmark detections per second, providing a significant quantity of data that could be used in the calculation of refined surgical performance metrics. The deep learning model had a detection accuracy of 85.9% when applied to hands wearing surgical gloves.

As a preliminary performance metric, we measured the time hands spent in each cell and the duration not actively engaged in the procedure, such as during instrument changes and microscope adjustments. Time spent in each cell may allow assessment of various aspects of procedures, such as evaluating the efficiency of instrument movements and changes, microscope adjustments, and overall workflow. This data can be used to optimize surgical techniques, reduce unnecessary movements, and improve the ergonomics of the surgical environment. The hand tracking system was used with gloves and darker lighting conditions simulating an operating room environment, providing preliminary evidence that this technology could be used to track hand positions during actual surgical procedures in the operating room.

Despite limitations such as tracking loss caused by microscope light and the model being trained for ungloved hands, we successfully captured and analyzed the tracking data. However, drilling involves a broader range of movements, from gross to micro, which introduces additional challenges. Future studies should examine if hand movements correlate with movements of the surgical instrument. To validate this method, it is necessary to compare groups where both the movement of the surgical instrument and the hand (as a surrogate for the instrument's movement) are evaluated. Additionally, we plan to analyze movement variability among different skill levels, including experts and trainees.

Many aspects of automatic detection of surgical hand and instrument movements will need to be explored and validated before such data can be translated and relied upon to guide or inform the actual human clinical or surgical scenario. We are exploring the use of multiple cameras to improve hand tracking by capturing positions from various angles. We are working with cadaveric models to replicate actual clinical operative maneuvers, with the aim of translating these findings into clinical studies. However, integrating multiple imaging nodes with ML-based data acquisition will require managing vast data sets. A multi-camera setup in the operating room, for example, could become overly complex. While more data may offer more significant insights, the goal should be to determine the minimal technology setup that provides practical, efficient, and meaningful insights for improving or monitoring surgical techniques, movement prediction, and outcomes. Implementing machine learning could provide more sophisticated methods to analyze a large volume of tracking data. Certainly, in this regard, the collaboration of bioengineers and data scientists with neurosurgeons is requisite.

## 5 Conclusions

Hand motion detection using a deep learning hand detector without physical sensors on the hands during cadaveric microsurgical mastoidectomy dissections is a feasible method to gather data on hand landmark position, assess surgical performance, and provide feedback to neurosurgical trainees. This method can be used to perform quantitative motion analysis of different surgical techniques. However, further studies are needed to develop more advanced metrics and evaluate the instructional value of this system in microsurgical training. With further development, this technology holds significant potential for enhancing the assessment and training of neurosurgical skills.

## Data Availability

The raw data supporting the conclusions of this article will be made available by the authors, without undue reservation.

## Supplementary material

Supplementary Video 1 Video demonstrating real-time deep learning hand motion detection during a cadaveric mastoidectomy procedure. This is a cropped segment of the full video. Used with permission from Barrow Neurological Institute, Phoenix, Arizona. © Barrow Neurological Institute, Phoenix, Arizona.
